# Adolescent deliveries in rural Cameroon: comparison of delivery outcomes between primipara and multipara adolescents

**DOI:** 10.1186/s13104-018-3550-z

**Published:** 2018-07-03

**Authors:** Tsi Njim, Valirie Ndip Agbor

**Affiliations:** 1 Centre for Global Health and Tropical Medicine, Nuffield Department of Medicine, University of Oxford, Old Road Campus, Oxford, Oxfordshire OX3 7BN UK; 2 Health and Human Development Research Group (2HD), Douala, Cameroon; 3 Ibal Sub-Divisional Hospital, Oku, North west Region Cameroon

**Keywords:** Adolescent deliveries, Multipara adolescents, Delivery outcomes, Cameroon

## Abstract

**Objective:**

Adolescent pregnancies are high risk and deliveries in this age group are usually associated with adverse outcomes. The perception that multiparous adolescents have better delivery outcomes than primiparous counterparts is not uncommon. We sought to determine if multiparous adolescents were precluded from having adverse delivery outcomes when compared to primiparous adolescents. The data used for the analysis is a side product from a published project aimed at mapping the epidemiology of adolescent deliveries in the Oku health district.

**Results:**

From an 8-year (2009–2016) retrospective register analysis of data from two primary healthcare facilities in the Oku health district—a rural area in Cameroon, the prevalence of multiparous adolescent deliveries was 21.5% (78/363). After multivariable analyses, and adjusting for age, sex of baby, gestational age, marital status and HIV status, primiparous adolescents were more likely to have low birth weight infants (LBW) (OR: 3.2; 95% CI 1.1, 9.7; p = 0.04) when compared with multiparous adolescents. Though primiparous adolescents were more likely to have LBW infants than multiparous adolescents, this group of mothers are generally ill-equipped to handle pregnancies and adolescent-friendly programs are necessary to decrease the associated burden.

**Electronic supplementary material:**

The online version of this article (10.1186/s13104-018-3550-z) contains supplementary material, which is available to authorized users.

## Introduction

Adolescent deliveries occur in girls aged 13–19 years [1]. As they occur at the stage of biological, psychological, and social transition in the adolescents’ lives [2], they are usually classified as high risk [3]. Compared with adult deliveries, the risk of adverse maternal and foetal outcomes in adolescent deliveries are higher. Indeed, several authors have shown an increased association of these deliveries with preeclampsia/eclampsia; operative deliveries; maternal and foetal death; foetal distress; low birth weight (LBW); neonatal asphyxia and stillbirth [2–5]. Such deliveries are a huge public health problem especially in the low-income countries where over 95% of them occur [6]. This poses major hindrance against the attainment of Sustainable Development Goal 3 [7]. The prevalence of adolescent deliveries in Cameroon is relatively high—with a national prevalence of 14.2% [3]. This prevalence is highest in rural areas of the country where early marriages are promoted, and sexual education and contraceptive use is low. The incidence of complications in such areas cannot be underestimated as optimal antenatal care and safe delivery procedures remain inadequate [8]. The situation is worsened by concepts that married adolescents or adolescents who have had a previous pregnancy ultimately have better outcomes than their single or primiparous counterparts. A recent study in a suburban region in the country showed that being married did not preclude an adolescent from having adverse pregnancy outcomes when compared with single adolescents [2]. This finding was also confirmed in the Oku health district from published data that was used in the analysis of the present study [9].

We sought to establish if being multiparous precluded an adolescent from having adverse delivery outcomes when compared with their primiparous counterparts in a rural setting—the Oku health district in Cameroon. Such findings could inform public policy for the implementation of health promotion strategies that could help reduce negative conceptions and reduce the burden associated with adolescent deliveries.

## Main text

### Research objectives

1. To determine the prevalence of multiparous adolescent pregnancies in a group of primiparous and multiparous adolescents in the Oku health district.
2. To compare the delivery outcomes between these primiparous and multiparous adolescents.

#### Study design, duration and setting

We carried out an 8-year retrospective-register analysis of maternity records from 1st January 2009 to 31st December 2016 in the Oku district hospital and Kevu primary health centre. These two primary health care facilities are the major health facilities in the Oku health district (OHD) and carry out most of the deliveries in this rural area.

**Fig. 1:**
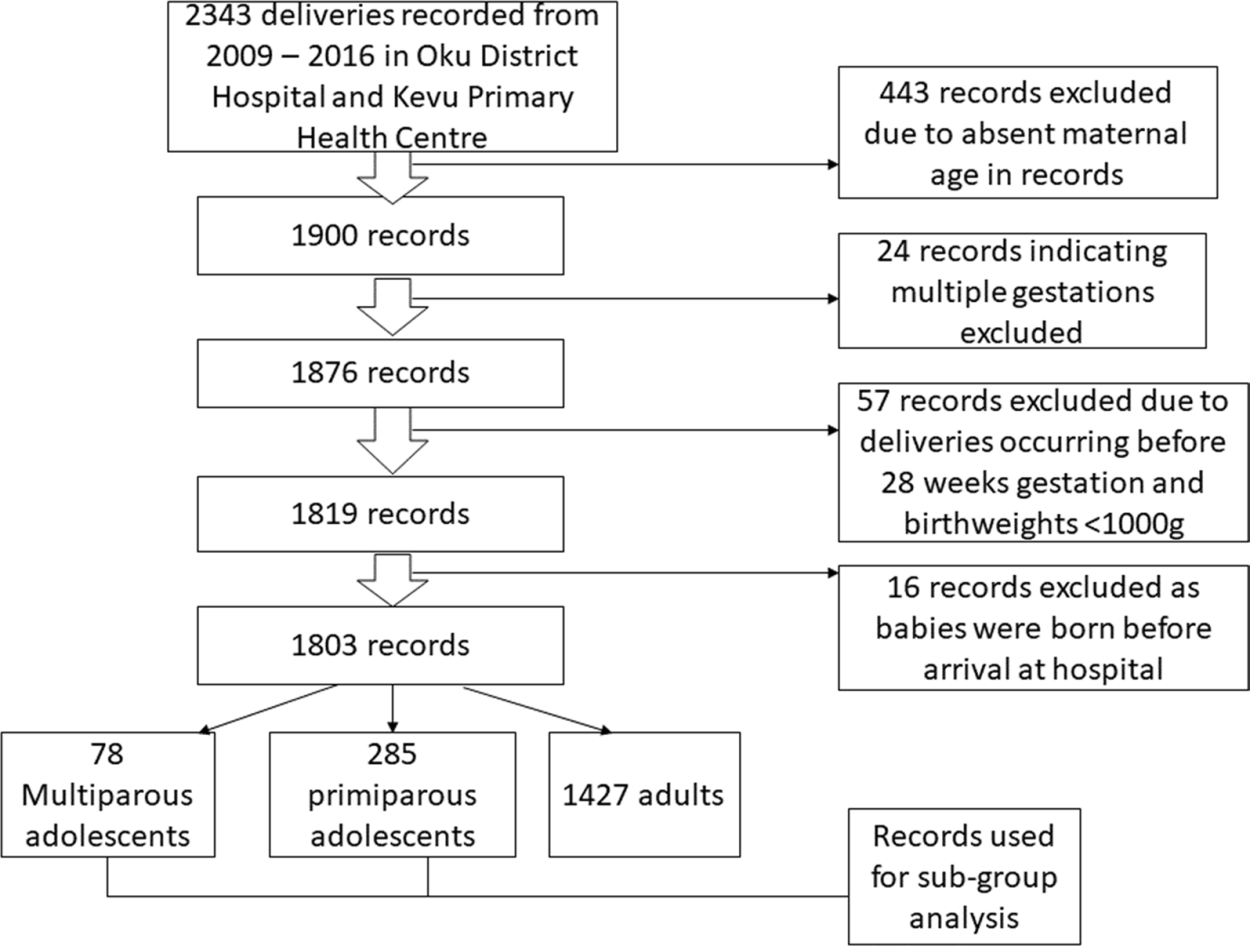
Flow diagram showing reasons for exclusion of records from analysis

The data collection procedure, inclusion and exclusion criteria have been described in a prior study [9]. All singleton deliveries recorded within the study period were included. Records: without maternal age, babies born before arrival, birthweights < 1000 g, multiple deliveries and deliveries before 28 weeks gestation were excluded from our study (Fig. 1).

From the 2343 deliveries that occurred during the 8-year study period, sociodemographic information (age, marital status), clinical characteristics (gravidity, parity, gestational age, human immunodeficiency virus [HIV] status and sex of the neonates), maternal outcome (mode of delivery, postpartum haemorrhage and second—fourth degree perineal tear) and foetal outcome (birthweight, fifth minute Apgar score and term gestational age were collected from 1803 records giving a response rate of 77%.

#### Statistical analysis

Data were entered using Epi info 7.0.8.3 software and analysed using STATA version 12.1 after verification of 10% of the data. Means (standard deviations) were used to summarize continuous variables and proportions for categorical variables. Means for variables that are not normally distributed were compared using the Kruskal–Wallis test. Proportions were compared using the Fisher exact test. Odds ratios and their corresponding 95% confidence intervals (CI) were used to describe the association between two categorical variables with significance obtained at 5%. Time trends were established using the Mann–Kendall test.

The 363 records eligible for subgroup analysis were split into primiparous and multiparous adolescents. Delivery outcomes were compared between the primiparous adolescents and the multiparous adolescents. A multivariable logistic regression analysis was performed for significant adverse delivery outcomes between primiparous and multiparous adolescents to adjust for potential confounders.

#### Ethical considerations

Ethics approval was obtained from the scientific and ethical review board of the Northwest Regional delegation for Public Health.

### Results

There were 363 adolescent deliveries with 21.5% (95% CI 17.6, 26.0) of them being multiparous adolescents. The prevalence of multiparous adolescent deliveries from 2009 (25.9%) fell to 15.8% in 2010 and rose steadily (20.6% in 2011, 22.7% in 2012 and 33.9% in 2013) before falling (19.3% in 2014, 15.9% in 2015 and 9.4% in 2016). The trend was however not significant (p = 0.3) (Additional file 1: Figure S1). The mean age of primiparous adolescents was 17.8 ± 1.3 while that for their multiparous counterparts was 18.3 ± 1.0 (p < 0.01). Fifty-nine percent of multiparous adolescents were married compared to 34.7% of primiparous adolescents (Table 1).

**Table 1:** Characteristics of adolescents from the Oku health district, 1st January 2009 to 31st December 2016

Characteristic	Primipara adolescents		Multipara adolescents		Adults	
	n = 285	%	n = 78	%	n = 1427	%
Marital status						
Married	186	65.3	32	41.0	220	15.4
Single	99	34.7	46	59.0	1207	84.6
HIV status						
Positive	6	2.1	5	6.4	70	4.9
Negative	279	97.9	73	93.6	1362	94.1
Sex of infant						
Male	149	52.5	30	38.5	729	51.0
Female	135	47.5	48	61.5	701	49.0

When compared with multiparous adolescents, primiparous adolescents were three times more likely to have a low birth weight (LBW) infant (OR: 3.3; 95% CI 1.1, 9.5; p = 0.01) (Table 2). After multivariable logistic regression analysis, being a primipa was the only independent predictor of having a LBW infant in adolescents (Additional file 2: Table S1).

**Table 2:** Pregnancy outcomes of deliveries in multiparous adolescents compared with nulliparous adolescents, 1st January 2009 to 31st December 2016

Delivery outcomes	Primiparas		Multiparas		Odds ratios	95% CI	p-value
	N 285	% (78.5)	N 78	% (21.5)			
Preterm delivery							
Yes	85	33.2	17	23.0	1.7	0.9–3.0	0.06
No	171	66.8	57	77.0			
Operative delivery^a^							
Yes	1	0.4	0	0.0	–	–	
No	283	99.6	78	100			
Perineal tears^b^							
Yes	24	8.4	8	10.3	1.2	0.5–2.9	0.4
No	261	91.6	70	89.7			
Low birth weight^c^							
Yes	43	15.5	4	5.3	3.3	1.1–9.5	0.01
No	235	84.5	72	94.7			
Neonatal asphyxia^d^							
Yes	28	10.0	10	13.2	0.7	0.3–1.6	0.3
No	251	90.0	66	86.8			
Stillbirth							
Yes	5	1.8	1	1.3	0.7	0.08–6.3	0.6
No	279	98.2	77	98.7			
Post-term deliveries^e^							
Yes	14	5.5	8	10.8	0.4	0.2–1.2	0.09
No	242	94.5	66	89.2			
High birth weight^f^							
Yes	7	2.5	2	2.6	1.0	0.2–4.7	0.6
No	271	97.5	74	97.4			

^a^Operative deliveries: Caesarean sections

^b^Perineal tears: second—fourth degree tears

^c^LBW: birth weights ≤ 2600 g [14]

^d^Neonatal asphyxia: fifth minute APGAR score < 8

^e^Post-term deliveries: deliveries at gestational age < 42 completed weeks

^f^High birth weight: birth weights ≥ 3850 g [15]

There was no difference in the following outcomes between primiparous and multiparous adolescent deliveries: preterm and post-term deliveries, operative deliveries, perineal tears, neonatal asphyxia, stillbirths and high birth weight (HBW) (Table 2).

### Discussion

In this study, we assessed if delivery outcomes differed between multiparous and primiparous adolescents. We observed a downward insignificant trend in the prevalence of multiparous adolescent deliveries from 2009 to 2016. The overall prevalence of multiparous adolescent deliveries over the 8-year period was 21.5%. Primiparous adolescents were more likely to have LBW neonates when compared with multiparous adolescents. In the prior study using this dataset, the prevalence of adolescent deliveries was 20.4% and the outcomes of adolescent deliveries compared with adult deliveries were: second—fourth degree perineal tears, low birth weight (LBW) and neonatal asphyxia at the fifth minute [9].

Throughout literature, only one study which tried to assess pregnancy outcomes between these two groups of adolescents was found. This was a study carried out in a semi-urban region—Bamenda in Cameroon and showed that adolescent primipas were just as likely to have LBW, HBW, asphyxia and caesarean sections as adolescent multipas [10]. The sample size used in this study was however small to get significant differences between the two groups. It is a common perception in most rural settings in Cameroon which encourage early marriages that multiparous or married adolescents are precluded from having complications when compared with primiparous or single adolescents. A prior study in a semi-urban population in the South west region of the country showed that delivery outcomes were similar between married and single adolescents [2]. From a prior study in this same region which used the same dataset, these findings were confirmed as outcomes were similar between married and single adolescents [9]. We showed that though primiparous adolescents were more likely to have LBW babies, most of the pregnancy outcomes (preterm deliveries, post-term deliveries, operative deliveries, neonatal asphyxia and stillbirths) occurred in similar proportions between these two groups. The sample size was however small to show significant differences between some of the outcomes. These hypotheses and findings should therefore be confirmed using a larger sample size. Also, the wide confidence intervals obtained for the association between being a primiparous adolescent and having a LBW neonate emphasises the need for more studies with robust sample sizes. Caution is therefore required when interpreting these results.

The fact that multiparous adolescents had a lower proportion of LBW infants could probably be explained by the hypothesis that these adolescents having had a previous pregnancy during which they perceived the benefits of antenatal care (ANC), thus, attended and received optimal ANC during the current pregnancy. Danger signs associated with LBW were therefore identified and managed. This hypothesis however should be tested in subsequent studies.

Though multiparous adolescent deliveries were precluded from having a single adverse outcome in this study—LBW, it should be noted that these group of adolescents are still likely to have adverse delivery outcomes when compared with adults as seen in the previous analyses [9].

Several authors have reported a high incidence of adverse outcomes in adolescents such as perineal tears, post-term deliveries and neonatal asphyxia. Perineal tears are a common finding in adolescent deliveries as reported by authors in Cameroon [2, 11, 12] while the babies born from adolescent mothers are usually prone to asphyxia [2, 13]; probably because of the prolonged labours that their mothers tend to have.

This analysis emphasises the fact that adolescents are generally not prepared to handle the burden of pregnancy [2, 3]. Preventive strategies that could reduce the rate of early marriages, adolescent pregnancies and its associated complications are therefore warranted to reduce this public health problem. These preventive strategies could include more adolescent-friendly programmes that encourage sexual health education and the use of contraceptives among adolescents. This should be accompanied by integration of contraceptive clinics in routine healthcare practices in health centres and outreach activities to schools targeting adolescents to promote sexual health.

### Conclusion

Though primiparous adolescents were more likely to have LBW neonates than their multiparous adolescents, this group is generally ill-equipped to deal with the burden of pregnancy. The hypothesis and findings generated in this study needs to be confirmed by future studies with robust designs.

## Limitations

Being a retrospective study, there is the potential limitation that there were errors in the registers, some key outcomes and exposures could not be measured and there is the risk of selection bias. However, this study generates an important hypothesis that needs to be supported by more robust designs like cohort studies in future projects guided by power and sample size calculations. The study was carried out in two health facilities in the Oku health district and does not necessarily reflect the entire situation in all rural areas of Cameroon.

## Additional files

**Additional file 1: Figure S1.** The prevalence of multiparous adolescent deliveries among adolescents in the Oku Health district over 8-year period from 2009 to 2016.

**Additional file 2: Table S1.** Multivariable logistic regression analysis showing the influence of parity on low birth weight after adjusting for confounders.

## Abbreviations

LBW: low birth weight
OHD: Oku health district
HIV: human immunodeficiency virus
CI: confidence intervals
HBW: high birth weight
ANC: antenatal care
