# Supplementary figures and images for: Adolescent deliveries in rural Cameroon: comparison of delivery outcomes between primipara and multipara adolescents
Source: BMC Res Notes. 2018 Jul 3;11:427. doi: 10.1186/s13104-018-3550-z (PMC6029040; doi:10.1186/s13104-018-3550-z)

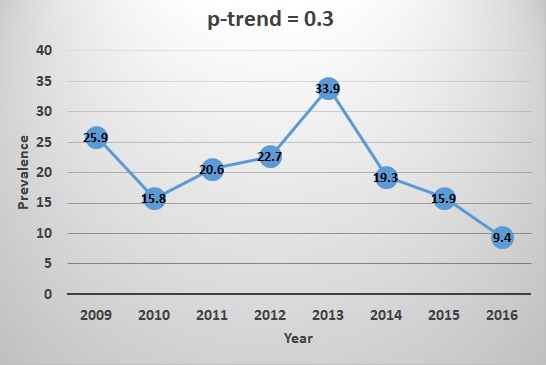

Supplement: Supplementary file 1 — Additional file 1: Figure S1. The prevalence of multiparous adolescent deliveries among adolescents in the Oku Health district over 8-year period from 2009 to 2016. [file 13104_2018_3550_MOESM1_ESM.jpg]
