# Supplementary material for: Adolescent deliveries in rural Cameroon: comparison of delivery outcomes between primipara and multipara adolescents
Source: BMC Res Notes. 2018 Jul 3;11:427. doi: 10.1186/s13104-018-3550-z (PMC6029040; doi:10.1186/s13104-018-3550-z)
Supplement: Supplementary file 2 — Additional file 2: Table S1. Multivariable logistic regression analysis showing the influence of parity on low birth weight after adjusting for confounders. [file 13104_2018_3550_MOESM2_ESM.docx]

| **Variable** | **Odds Ratio** | **95% Confidence intervals** | **p value** |
| --- | --- | --- | --- |
| **Primipa (Yes/No)** | 3.2 | 1.1, 9.7 | 0.04 |
| **Age (in years)** | 1.0 | 0.8, 1.4 | 0.82 |
| **Marital status (Married/Single)** | 0.8 | 0.4, 1.7 | 0.57 |
| **HIV status (Positive/Negative)** | 3.7 | 0.8, 16.3 | 0.09 |
| **Gestational age (in completed weeks)** | 1.0 | 0.9, 1.1 | 0.65 |
| **Sex of infant (Female/Male)** | 1.0 | 0.5, 2.1 | 0.89 |
